# A Novel Reduplicate Strategy for Tracing Hemostatic Compounds from Heating Products of the Flavonoid Extract in *Platycladi cacumen* by Spectrum-Effect Relationships and Column Chromatography

**DOI:** 10.3390/molecules200916970

**Published:** 2015-09-17

**Authors:** Yeqing Chen, Hongli Yu, Hao Wu, Yaozong Pan, Kuilong Wang, Liping Liu, Yangping Jin, Chengchao Zhang

**Affiliations:** 1 School of pharmacy, Nanjing University of Chinese Medicine, Nanjing 210023, China; E-Mails: cyqzs88@163.com (Y.C.); yuhongli76@126.com (H.Y.); pan_yaozong@126.com (Y.P.); wjwkl@126.com (K.W.); liuliping1487@163.com (L.L.); yangpingctt@126.com (Y.J.); didiaodexiaojuren@163.com (C.Z.); 2 Jiangsu Key Laboratory of Chinese Medicine Processing, Nanjing University of Chinese Medicine, Nanjing 210023, China; 3 Engineering Center of State Ministry of Education for Standardization of Chinese Medicine Processing, Nanjing 210023, China

**Keywords:** flavonoids, heating products, hemostatic activity, *Platycladi cacumen*, spectrum-effect relationships

## Abstract

*Platycladi cacumen* and its processed product have been utilized as a Chinese medicine to treat hemorrhages. In this study, the base peak chromatogram fingerprints of heating products of total flavonoids in *Platycladi cacumen* were established by high performance liquid chromatography coupled with mass spectroscopy/mass spectroscopy (HPLC-MS/MS), and the hemostatic activities were studied by hemostatic screening tests *in vivo*. The spectrum-effect relationships between fingerprints and hemostatic activities were analyzed by using canonical correlation analysis to trace the peaks responsible for the significant hemostatic effects. Peak 10 and peak 12 were correlated most closely, thus probably being the main hemostatic compounds. To confirm the reliability of this strategy, the targeted unknown peak was obtained by bioactivity-guided isolation, characterized by MS, ^1^H-NMR, ^13^C-NMR, and 2D-NMR spectroscopies, and referred to as cecarbon as a new compound. In addition, the isolated compound exhibited hemostatic effect in a dose-dependent manner with different potencies *in vitro* and existed in *Platycladi cacumen Carbonisatus*. A novel dereplication strategy was employed to trace and identify the active compounds of other herbs that have bioactivity enhancement after processing using spectrum–effect relationships and column chromatography.

## 1. Introduction

As a traditional Chinese medicine (TCM), *Platycladi cacumen* (PC) is the dried branches and leaves of *Platycladus orientalis* (L.) Franco, which belongs to the monotypic genus *Platycladus* of the Cupressaceae family and has officially been listed in the Chinese Pharmacopoeia [1]. PC has been recorded and summarized in ancient manuscripts, including “Shu Ben Cao Ri Huazi Ben Cao”, “Ben Cao Bei Yao” and “De Pei Ben Cao” PC is mainly capable of removing heat from the blood, as well as preventing and treating hemorrhage diseases including hematemesis, epistaxis, hemoptysis, hematochezia, dysfunctional uterine bleeding, and hemorrhoidal bleeding [1,2]. It has been used in contemporary Chinese herbal formulations or dispensed as a medicine, such as Ce Bo San. Phytochemistry research found that flavonoids such as myricetrin, myricetin, quercitrin, quercetin, hinokiflavone, and amentoflavone are the main medicinal components of PC [3,4,5].

Carbonisatus product is produced by putting original species into a heating container to be processed into a burned black or burned brown surface and a brown or pale brown interior. It has been widely used to treat bleeding diseases. *Platycladi cacumen Carbonisatus* (PCC), which is the legal processed product of PC [1], has obviously weakened anti-blood-heat activity and enhanced anti-hemorrhagic activity, as suggested by pharmacological and clinical studies [6]. With styptic properties, PCC is commonly used in clinical practice to prevent and to treat various types of hemorrhages including hematochezia, hematemesis, epistaxis, dysfunctional uterine bleeding, and hemorrhoidal bleeding [1]. In addition, PCC has been applied to develop hemostatic agents (e.g., Shi Hui San). Unlike PC, flavonoid aglycones are the main effective components of PCC [7,8]. Despite ongoing endeavors, the components of PCC that show significant hemostatic activity remain unknown.

In this study, we hypothesized that therapeutic effects of PCC can be attributed to certain pharmacological and chemical correlations, particularly transformation of flavonoids during heating. Thus, it is of great significance to combine component analysis with structural identification for the simulated processing (heating) products of total flavonoids in *Platycladi cacumen* (FPC-N) showing hemostatic effect. However, it is too complicated to predict the activity of each chemical component, and the separation processes are difficult. Therefore, it is important to establish a reasonable spectrum-effect relationship model to analyze and associate the variety of effects caused by the transformations of chemical substances after processing.

The highly sensitive mass spectrometer (MS), which is often used as the mass analyzer for liquid chromatography (LC), facilitates the detection of low-content compounds that are not detectable using classical methods [9,10]. Recently, high-performance liquid chromatography coupled with mass spectroscopy/mass spectroscopy (HPLC-MS/MS) has been applied to rapidly analyze complex components and chemical transformations [11]. In particular, accurate masses and molecular formulae of untargeted compounds acquired from HPLC-MS/MS, as the most important information, have been used to predict and find the known components [12]. Hemostatic screening tests are classical and common in the laboratory [13], detecting bleeding time (BT), activated partial thromboplastin time (APTT), prothrombin time (PT), thrombin time (TT), fibrinogen content (FIB), euglobulin lysis time (ELT), and platelet aggregation. HPLC-MS/MS [14,15] and hemostatic screening tests [16,17], although widely used alone, have never been combined to analyze the active components in PC and PCC.

The aim of this study was to chemically and biologically characterize hemostatic compounds derived from FPC-N to reveal the material basis for the significantly enhanced hemostatic activity. The chromatograms and spectroscopic data acquired from HPLC-MS/MS were used to establish the fingerprints of FPC-N and to monitor changes on the chemical level during processing. Hemostatic screening tests were selected to evaluate the effects of these samples on blood *in vivo*. Some quantitative parameters obtained from the hemostatic activities were analyzed using principal component analysis (PCA). Then, the spectrum-effect relationships, combined with HPLC-MS/MS fingerprints and hemostatic activities of different samples, were investigated to trace the peaks responsible for the hemostatic effects, with the help of a canonical correlation analysis (CCA) model. Furthermore, the predicted active peaks in fingerprints were isolated by column chromatography (CC) and their structures were identified by MS, ^13^C- and ^1^H- nuclear magnetic resonance (NMR), two-dimensional NMR (2D-NMR), and ultraviolet (UV) spectroscopies. Finally, the hemostatic effect of the isolated compound was verified *in vitro*.

## 2. Results and Discussions

### 2.1. HPLC-MS/MS Analysis

**Figure 1:**
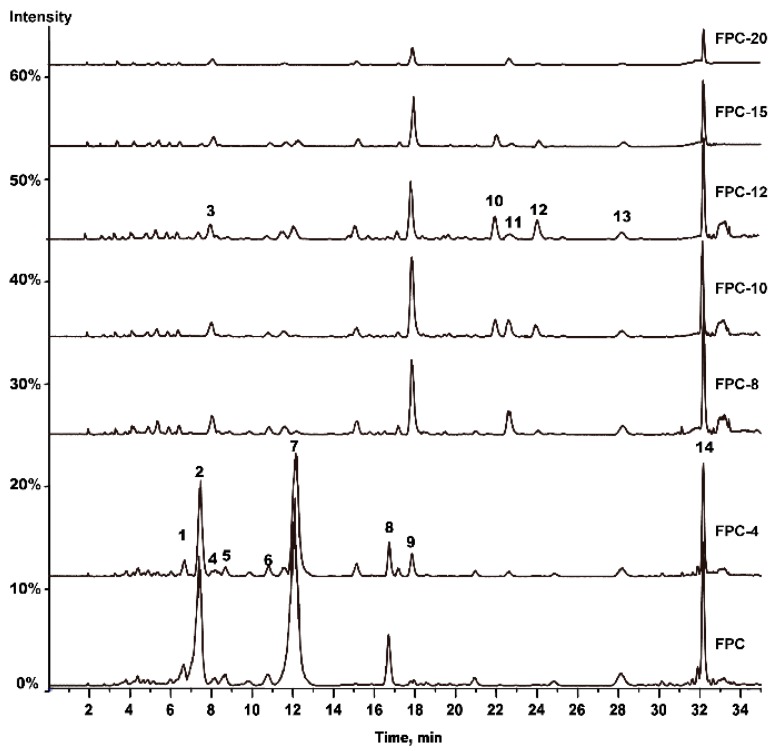
Representative BPC of FPC-N and its six processed products based on HPLC-MS/MS. A total of 14 peaks with large areas and good separation were regarded as “common peaks” in the fingerprints.

FPC-N samples were comparatively analyzed using our established HPLC-MS/MS method. The representative base peak chromatograms (BPC) of FPC-N are shown in Figure 1. Fourteen chromatographic peaks were detected and 10 peaks were identified by comparing their retention time and mass spectra with those of reference substances (Table 1). Notably, the relative peak areas of peaks 1, 2, 4–8, and 13–14 decreased with extended heating time. Furthermore, some substances were generated at different heating times, such as peaks 10 and 12, generated observably in FPC-10, and peak 11, generated in FPC-8. The relative heights of peaks 10–12 first increased and then decreased. The relative peak area of each peak that was obtained from XICs is shown in Table 2. Therefore, these seven FPC-N samples, as suggested by the 14 different characteristic peaks, showed significant differences.

**Table 1:** Characteristics of 14 peaks from FPC-N by HPLC-MS/MS in negative mode.

Peak No.	t_R_/min	[M − H]^−^ (Error (ppm))	Fragments	Formula	Identification
1	6.62	509.2050 (2.4)	491.1946, 367.1411, 313.1293, 179.0705	C_25_H_34_O_11_	Unknown
2	7.40	463.0864 (1.9)	316.0209, 287.0176, 271.0227, 178.9966	C_21_H_20_O_12_	Myricetrin
3	8.00	363.0731 (0.3)	331.0469, 300.0278, 271.0250, 178.9979, 151.0036	C_17_H_16_O_9_	Unknown
4	8.17	551.2191 (1.3)	505.2148, 341.1391, 329.1398, 314.1142	C_27_H_36_O_12_	Lyoniside
5	8.71	463.0894 (1.9)	301.0273, 271.0240, 255.0292, 151.0034	C_21_H_20_O_12_	Isoquercitrin
6	10.85	431.0999 (1.4)	311.0590, 268.0378, 240.0417	C_21_H_20_O_10_	Apigetrin
7	12.10	447.0922 (1.3)	301.0336, 271.0234, 255.0283, 151.0025	C_21_H_20_O_11_	Quercitrin
8	16.70	431.0984 (1.4)	284.0319, 255.0290, 227.0337	C_21_H_20_O_10_	Kaempferol-3-*O*-Rhamnoside
9	17.82	301.0349 (0.4)	273.0403, 178.9980, 151.0037, 121.0300, 107.0149	C_15_H_10_O_7_	Quercetin
10	21.90	411.0737 (1.0)	383.0778, 327.0515, 261.0410, 177.0188, 163.0034	C_21_H_16_O_9_	Huaicarbon B
11	22.60	285.0410 (−0.8)	239.0348	C_15_H_10_O_6_	Kaempferol
12	24.05	327.0718 (0.9)	312.0548, 281.0813, 268.0737, 211.0609, 165.0683	C_19_H_20_O_5_	Unknown
13	28.03	537.0853 (2.6)	417.0628, 399.0524, 375.0514, 331.0614, 257.0089, 117.0347	C_30_H_18_O_10_	Unknown
14	32.12	537.0836 (0.6)	443.0393, 417.0602, 399.0493, 375.0498, 331.0594	C_30_H_18_O_10_	Amentoflavone

**Table 2:** Relative peak area of each peak of FPC-N.

No.	t_R_/min	Average Peak Area of Every Peak							
		FPC	FPC-4	FPC-8	FPC-10	FPC-12	FPC-15	FPC-20	C.V. (%)
1	6.62	639,370	430,101	4746	0	0	0	0	87.68
2	7.40	3,673,429	2,305,206	16,025	0	0	0	0	93.86
3	8.00	12,221	147,248	202,203	177,952	173,135	85,047	47,777	91.28
4	8.17	234,301	176,238	27,590	16,045	11,729	11,904	0	101.10
5	8.71	209,800	154,003	30,984	0	0	0	0	92.84
6	10.85	301,206	170,929	27,102	17,140	15,029	14,914	2146	156.31
7	12.10	4,887,454	2,738,900	3,083,130	0	0	0	0	70.39
8	16.70	1,462,801	843,852	15,020	80,910	33,329	14,914	0	136.94
9	17.82	30,083	580,771	762,249	944,292	606,363	426,517	135,660	59.55
10	21.90	0	0	5168	212,541	260,343	102,742	1724	136.94
11	22.60	14,020	129,650	243,812	40,920	4630	2033	0	155.03
12	24.05	0	0	46,391	180,799	307,302	160,890	17,284	136.94
13	28.03	250,403	264,008	220,556	189,223	142,143	112,482	96,467	59.54
14	32.12	3,916,454	2,685,583	1,468,588	1,176,757	1,080,712	577,617	280,981	59.55

### 2.2. Hemostatic Activity

#### 2.2.1. Quantitative Hemostatic Parameters of FPC-N

The hemostatic activity was evaluated by measuring BT, APTT, PT, TT, FIB, ELT, and platelet aggregation *in vivo*. After administration, the hemostatic parameters were measured (Table 3). One-way analysis of variance (ANOVA) based on Student’s two-tailed unpaired t-test or Dunnett’s test for multiple comparisons test *vs.* control group was used for data analysis. Compared with the blank control group, the FPC-10, FPC-12, and FPC-15 groups significantly reduced BT, PT, and APTT and promoted collagen-induced platelet aggregation. However, TT, FIB, ELT, ADP-induced, and thrombin-induced platelet aggregation were similar in all the experimental groups, suggesting that only the products of FPC, after being heated from 10 to 15 min, had hemostatic effects that facilitated collagen-induced platelet aggregation and plasma coagulation. Even so, the parameters changed following different trends, making it difficult to accurately differentiate the effects of FPC-N on hemostatic activity. Therefore, it was necessary to find out the main parameter(s) that played the most important role in differentiating the FPC-N samples, for which PCA can be used.

**Table 3:** Hemostatic parameters of rat plasma affected by FPC-N.

Products	Parameters								
	BT(min)	PT (s)	APTT (s)	TT (s)	FIB (g/L)	ELT (min)	Maximal Aggregation (%)		
							ADP (5 μmol/L)	Collagen (0.3 μg/mL)	Thrombin (0.1 u)
Control	4.96 ± 0.56	9.87 ± 0.83	24.77 ± 0.61	16.88 ± 0.52	5.21 ± 0.47	100.08 ± 8.00	48.80 ± 3.65	40.35 ± 3.92	57.90 ± 4.02
YNBY	2.53 ± 0.35 **	7.41 ± 0.53 **	17.38 ± 0.64 **	16.63 ± 0.39	5.18 ± 0.32	98.50 ± 10.10	70.52 ± 4.15 **	52.24 ± 4.08 **	76.20 ± 3.82 **
FPC	4.86 ± 0.35	9.77 ± 0.46	24.59 ± 0.56	16.74 ± 0.46	5.24 ± 0.36	105.05 ± 9.35	30.68 ± 3.82 **	29.42 ± 3.05 **	33.95 ± 3.66 **
FPC-4	4.72 ± 0.34	10.10 ± 0.37	24.62 ± 0.51	16.59 ± 0.50	5.27 ± 0.34	96.85 ± 8.00	35.25 ± 4.28 **	34.29 ± 3.92 *	40.50 ± 3.78 **
FPC-8	4.50 ± 0.43	9.53 ± 0.39	24.03 ± 0.30	16.92 ± 0.61	5.28 ± 0.49	103.60 ± 7.85	42.35 ± 3.90	44.15 ± 4.20	51.35 ± 3.06
FPC-10	3.63 ± 0.41 **	8.88 ± 0.55 *	21.21 ± 0.54 **	16.53 ± 0.43	5.34 ± 0.40	99.05 ± 10.50	48.50 ± 4.15	49.51 ± 3.88 *	56.25 ± 4.08
FPC-12	2.98 ± 0.65 **	7.32 ± 0.63 **	19.38 ± 1.00 **	16.49 ± 0.43	5.29 ± 0.43	96.25 ± 9.90	50.25 ± 3.85	56.32 ± 3.75 **	52.07 ± 3.65
FPC-15	4.06 ± 0.45 **	8.80 ± 0.61 *	22.83 ± 0.52 *	16.34 ± 0.49	5.14 ± 0.36	101.55 ± 9.40	48.50 ± 3.82	46.29 ± 4.10	57.48 ± 3.92
FPC-20	4.86 ± 0.49	9.38 ± 0.48	23.83 ± 0.39	16.54 ± 0.45	5.19 ± 0.33	97.80 ± 8.45	47.95 ± 4.03	43.82 ± 3.41	55.05 ± 4.00

Data represents mean ± SD (*n* = 8). The negative control group (Control) was given blank solvent and the positive control group (YNBY) was treated with Yunnan Baiyao. ** *p* < 0.01 *vs.* control group; * *p* < 0.05 *vs.* control group.

#### 2.2.2. Results of PCA

PCA was used to find out the main parameters that played the most important role in differentiating the FPC-N samples; PCA was used in this part. Nine parameters (BT, PT, APTT, TT, FIB, ELT, ADP-induced, collagen-induced, and thrombin-induced aggregation) of the FPC-N samples were considered as objects and expressed as X_1_–X_9_. The PCA results showed that the first three principal components (Z_1_, Z_2_ and Z_3_) accounted for 87.06% of the cumulative contribution rate. The equations of the three principal components from the results of “Component Matrixa” are expressed as follows:

Z_1_ = −0.888X_1_ − 0.906X_2_ − 0.917X_3_ − 0.587X_4_ + 0.227X_5_ − 0.590X_6_ + 0.756X_7_ + 0.961X_8_ + 0.595X_9_ (1)

Z_2_ = 0.399X_1_ + 0.218X_2_ + 0.357X_3_ − 0.131X_4_ − 0.723X_5_ + 0.010X_6_ + 0.591X_7_ + 0.103X_8_ + 0.719X_9_ (2)

Z_3_ = 0.036X_1_ + 0.114X_2_ + 0.040X_3_ + 0.715X_4_ − 0.513X_5_ + 0.185X_6_ + 0.252X_7_ + 0.204X_8_ + 0.333X_9_ (3)

The absolute values before X_1_, X_2_, X_3_, and X8 in Z_1_, Z_2_, and Z_3_ are larger, showing that BT, PT, APTT, and collagen-induced aggregation play more important roles in differentiating the FPC samples. Besides, collagen-induced aggregation, which contributes more than BT, PT, and APTT do, was selected to compare the hemostatic effect of FPC-N. As a result, the activities of the tested groups followed a descending order of FPC-12 > FPC-10 > FPC-15 > the other groups that had no significant differences from each other. This might explain why the hemostatic effect of PCC surpassed that of PC and why PCC was required to retain characteristics to some extent.

### 2.3. Results of CCA

CCA was used for the spectrum-effect relationships between the areas of 14 peaks in the TICs and the main hemostatic activity parameters (BT, PT, APTT, and collagen-induced aggregation) (Table 4). The main hemostatic parameters were closely correlated with peaks 10 and 12 in the chemical spectra, *i.e*., these two peaks might be the main active components of FPC-N affecting hemostatic activity. Peak 10 (huaicarbon B) has been reported to show significant hemostatic activity [18], but further study was needed to identify the structure and confirm the bioactivity of predicted active peak 12.

**Table 4:** Correlation coefficients between chromatograms peaks and main hemostatic parameters.

**Parameters**	**Peak No. (1−7)**						
	**1**	**2**	**3**	**4**	**5**	**6**	**7**
BT	0.5368	0.5332	−0.5557	0.5262	0.5732	0.5116	0.6068
PT	0.5782	0.5680	−0.3176	0.5952	0.6285	0.5478	0.6507
APTT	0.5695	0.5630	−0.4571	0.5756	0.6202	0.5469	0.6778
Collagen-Aggregation	−0.8849	−0.8822	0.5644	−0.8828	−0.9026	−0.8702	−0.8286
**Parameters**	**Peak No. (8–14)**						
	**8**	**9**	**10**	**11**	**12**	**13**	**14**
BT	0.5158	−0.5721	−0.9767	0.2719	−0.9861	0.3400	0.4023
PT	0.5423	−0.2718	−0.8879	0.4365	−0.9588	0.5757	0.5044
APTT	0.5434	−0.4706	−0.9784	0.3901	−0.9729	0.4494	0.4658
Collagen-Aggregation	−0.8703	0.5485	0.8170	−0.1752	0.8742	−0.6576	−0.7950

### 2.4. Isolation and Confirmation of the Predicted Active Peaks

#### 2.4.1. Isolation and HPLC Analysis

**Figure 2:**
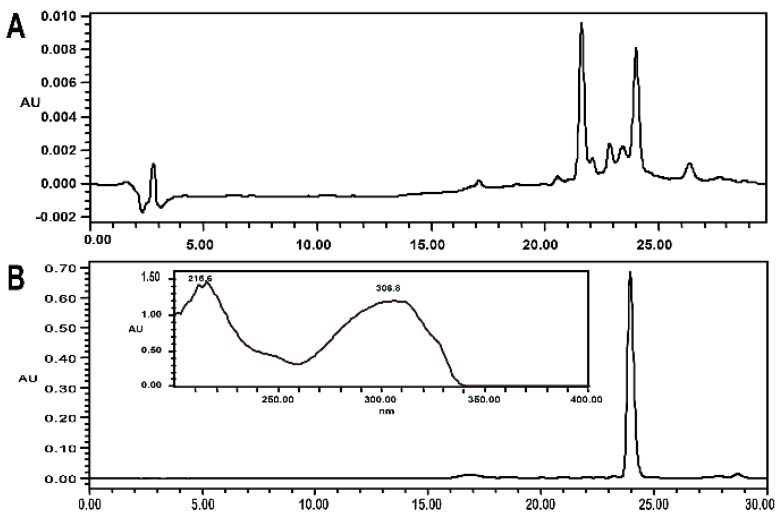
HPLC chromatogram of the fraction containing the peak 12 (**A**); HPLC chromatogram and UV spectrum of the peak 12 (**B**).

The eluates containing target peak 12 and bioassay-guided fractions containing peak 12 in FPC-12 extract were further separated by column chromatography (CC), giving the purified compound (6.2 mg, purity >92%). The purity analysis results are shown in Figure 2.

#### 2.4.2. Structural Identification

The chemical structure of the targeted compound (peak 12) was identified according to the MS, 1D-NMR, and 2D-NMR data.

**Figure 3:**
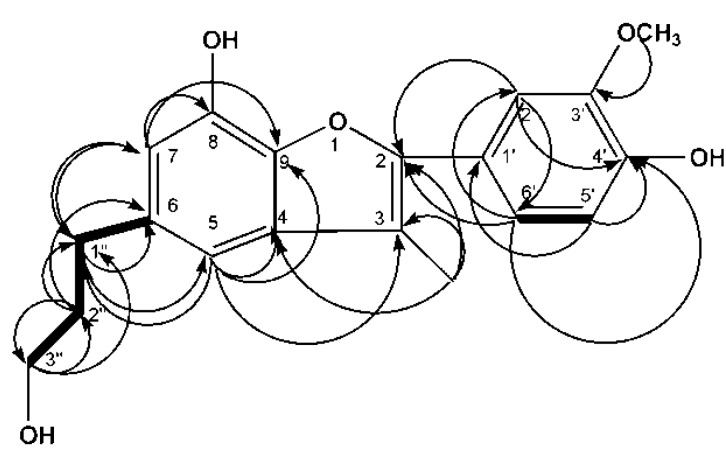
Key HMBC (→) and COSY (−) correlations of cecarbon.

**Figure 4:**
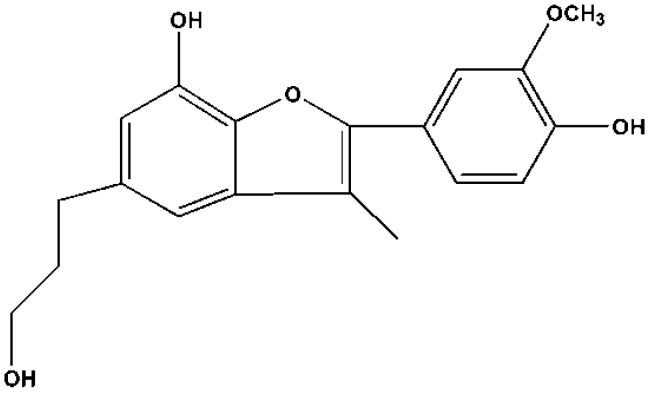
Structure of cecarbon.

The molecular formula of peak 12 was determined to be C_19_H_20_O_5_ on the basis of the molecular ion at *m*/*z* 327.1235 [M − H]^−^ by HR-EI-MS. As evidenced by ^1^H-NMR, ^13^C-NMR and HSQC spectra (Table 5), it contained a methoxyl group, a methyl group, three methylene groups, and five methine groups. DEPT, ^1^H-^13^C HMBC, and ^1^H-^1^H COSY were also performed to determine protons linked to individual carbons. HMBC analysis (Figure 3) was employed to confirm the correlation between methylene protons at δ_H_ 2.63, δ_H_ 1.75, δ_H_ 3.44 and the quaternary carbon at δ_C_ 137.28, supporting the linkage of the group to C-6. Methyl protons show HMBC with C-1′, C-3, C-2, and C-4, indicating that the group was connected to C-3. The COSY spectrum (Figure 3) shows coupling between methylene protons at δ_H_ 1.75 and those at δ_H_ 2.63 and δ_H_ 3.44. A similar shift concerning a similar compound has been reported before by Kimura *et al.* [19]. By searching databases such as SciFinder Scholar of American Chemical Society, peak 12 was discovered to be a new compound and designated as 2-(4-hydroxy-3-methoxy-phenyl)-5-(3-hydroxyl-propyl)-3-methyl-benzofuran-7-ol, referred to as cecarbon. Their structures are shown in Figure 4.

**Table 5:** ^1^H- and ^13^C-NMR data of peak 12.

Position	δ_H_, mult., *J* (Hz)	δ_C_		Position	δ_H_, mult., *J* (Hz)	δ_C_	
1				2′	7.29 s	110.32	CH
2	-	150.32	C	3′	-	147.74	C
3	-	109.24	C	3′-OCH_3_	3.84	55.68	CH_3_
3-CH_3_	2.36 d (4.5)	9.29	CH_3_	4′	-	146.86	C
4	-	132.61	C	4′-OH	9.60	-	-
5	6.82	111.09	CH	5′	6.93 d (5.1)	115.81	CH
6	-	137.28	C	6′	7.22 dd (6, 3.9)	119.59	CH
7	6.58	108.89	CH	1′′	2.63 t (9.0)	31.81	CH_2_
8	-	141.63	C	2′′	1.75 m (17.1)	34.81	CH_2_
8-OH	9.56	-	-	3′′	3.44 t (7.5)	60.17	CH_2_
9	-	140.04	C	3′′-OH	4.41 s	-	-
1′	-	122.28	C				

s, singlet; d, doublet; dd, doublet of doublet; m, multiplet; t, triplet.

#### 2.4.3. Hemostatic Activity Verification

**Figure 5:**
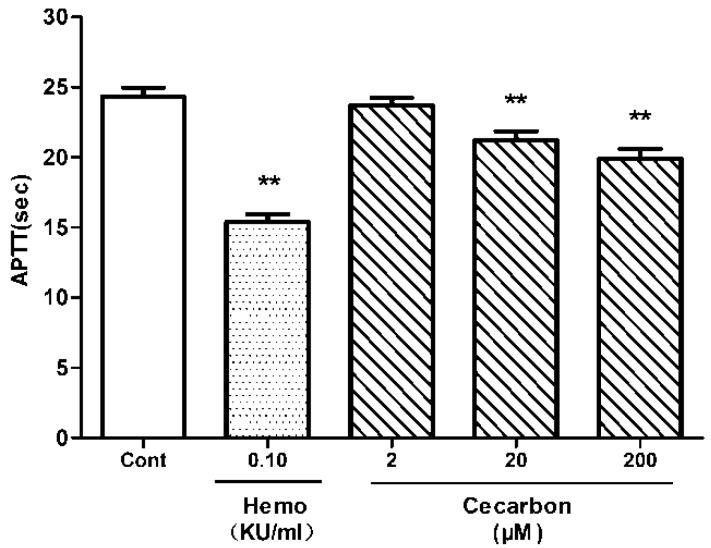
Effect of cecarbon on New Zealand rabbit plasma APTT. Data were expressed as mean ± SD (*n* = 6) and *p*-values were calculated using non-parametric repeated measures ANOVA (Friedman test) (** *p* < 0.01).

**Figure 6:**
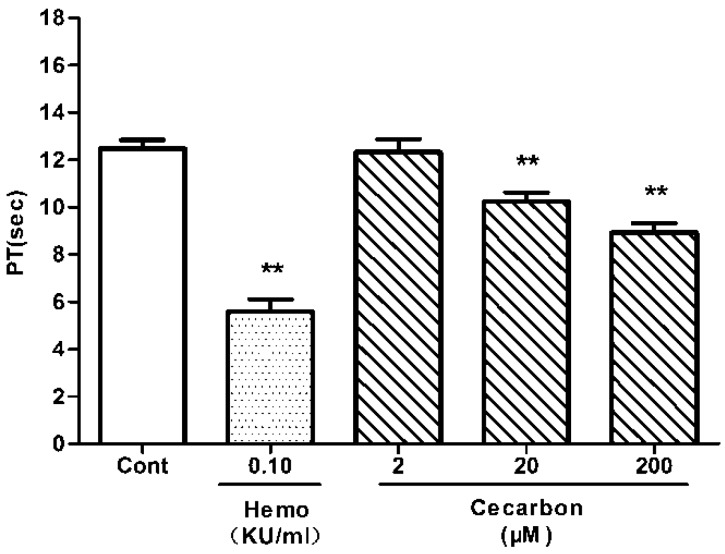
Effect of cecarbon on New Zealand rabbit plasma PT. Data were expressed as mean ± SD (*n =* 6). The *p*-values shown were calculated by using non-parametric repeated measures ANOVA (Friedman test) (** *p* < 0.01).

**Figure 7:**
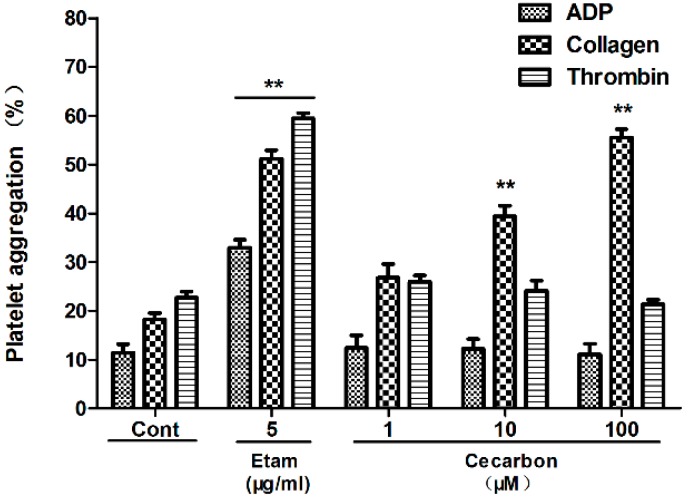
Effect of cecarbon on New Zealand rabbit platelet aggregation induced by ADP, collagen and thrombin. Data were expressed as mean ± SD (*n =* 6). The *p*-values were calculated by non-parametric repeated measures ANOVA (Friedman test) (** *p* < 0.01).

The hemostatic activity of peak 12 was investigated *in vitro* by measuring coagulation parameters (APTT, PT) and the platelet aggregation induced by various agonists. Peak 12 dose dependently decreased APTT (Figure 5) and PT (Figure 6). As shown in Figure 7, the compound promotes collagen-induced platelet aggregation in a dose-dependent manner, but does not significantly affect that induced by ADP or thrombin. These results agreed well with the prediction. Hence, peak 12 in the BPC fingerprints was indeed one of the hemostatic compounds of FPC-N.

#### 2.4.4. Application to the Analysis of PCC Samples

**Figure 8:**
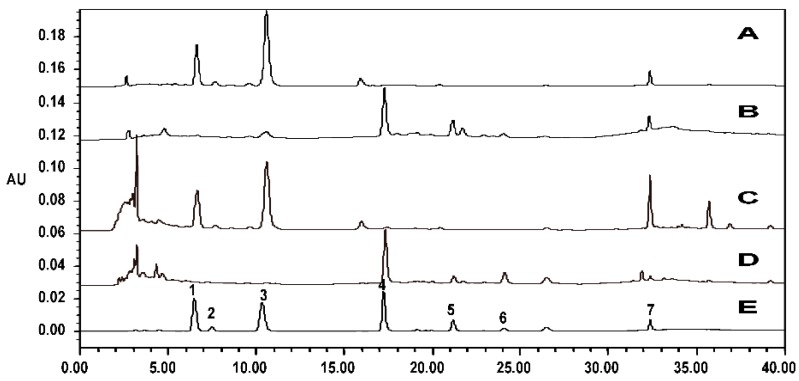
HPLC chromatogram of sample FPC (**A**); sample FPC-12 (**B**); sample PC (**C**); sample PCC (**D**); and the standard mixtures (**E**). Seven peaks were identified by comparing with the standard substance: myricetrin (1), isoquercitrin (2), quercitin (3), quercetin (4), huaicarbon B (5), cecarbon (6), and amentoflavone (7).

In TCM practice, PC is processed (heated) in the form of whole branches and leaves rather than a flavonoid extract. To verify whether the resultant new compound obtained from this study is the legitimate active compound formed after PC is processed/heated in practical TCM, the chemical compositions of PCC extract were detected using HPLC. The hemostatic compound was found in PCC (Figure 8). Furthermore, the chemical compositions of FPC-12 were similar to those of PCC extract. Therefore, the subjects of this study were appropriately selected to reflect the reality of TCM practice for PC processing.

## 3. Experimental Section

### 3.1. Materials and Reagents

Fresh branches and leaves of *Platycladus orientalis* (L.) Franco were collected from Heibei Province, China, identified by Professor Chungen Wang (College of Pharmacy, Nanjing University of Chinese Medicine), and then dried. Quercitin, quercetin, myricetrin, afzelin, isoquercitrin, lyoniside, amentoflavone, kaempferol, and apigetrin with 98% purity were purchased from the National Institutes for Food and Drug Control (Beijing, China). Huaicarbon B with 93% purity was provided by the Jiangsu Key Laboratory of Chinese Medicine Processing, Nanjing University of Chinese Medicine, China. HPLC-grade methanol and formic acid were purchased from Merck (Darmstadt, Germany). Water for HPLC was purified with a purifier (Research Scientifics Instruments Co. Ltd., Xiamen, China). All solvents used for extraction and CC separation were of analytical grade. Sephadex LH-20 for CC was purchased from GE Healthcare Bio-Science AB. (Uppsala, Sweden), and SiliaSphere PC C18 for CC was from SiliCycle Inc. (Quebec, QC, Canada). Semi-coagulation analyzer (Model LG-PABER-I) was purchased from Steellex Scientific Instrument Company (Beijing, China). A coagulation assay kit was purchased from Nanjing Jiancheng Bioengineering Institute (Nanjing, China). ADP, collagen, and thrombin were purchased from Sigma (St. Louis, MO, USA).

### 3.2. HPLC-MS/MS

#### 3.2.1. Sample Extraction and Preparation

FPC was obtained first to prepare FPC-N. PC was extracted with 65% ethanol (15:1, *v*/*w*) for 2 h, after which the solution was filtered and concentrated to dryness by evaporation to obtain the FPC with 90% purity (measured by HPLC, the result was provided in Supplementary Materials Figure S1 and Table S1). FPC-N was prepared using single component heating (250 °C) that simulated the carbonization process. FPC (1.5 g, 100 g crude herb × 1.41% total flavonoid content)/90% purity) powders were put into a 100-mL crucible and heated in a muffle furnace for 4 min (FPC-4), 8 min (FPC-8), 10 min (FPC-10), 12 min (FPC-12), 15 min (FPC-15), and 20 min (FPC-20), respectively, to obtain FPC-N.

The FPC-N samples were ground to fine powders in a mill. Accurately weighed powders (10 mg) were dissolved with 10 mL of methanol, and sonicated for 30 min. The supernatant was filtered through a 0.22-μm syringe filter before being analyzed. Fingerprints were established based on the peak areas and retention times of the main components in FPC-N.

#### 3.2.2. Instruments and HPLC-MS/MS Conditions

HPLC-MS/MS analysis was performed on 20DXR HPLC system (SHIMADZU, Japan). Separation was performed with a Dubhe C18 column (250 mm × 4.6 mm, 5.0 mm; Hanbon, China). The mobile phase was a mixture of methanol (A) and 0.1% formic acid-water (B), with an optimized linear gradient elution as follows: 0–10 min: 48% A; 10–15 min: 48%–60% A; 15–25 min: 60% A; 25–30 min: 60%–80% A; 30–40 min: 80% A. The flow rate was 1.0 mL/min. The injection volume was 10 μL. The flow rate was set at 1.0 mL/min with split ratio of 1:1. The column temperature was set at 30 °C.

Mass spectrometry was performed on the Triple TOF™ 5600 equipped with an electrospray ionization (ESI) source (AB SCIEX, Redwood City, CA, USA). Samples were analyzed in negative ion modes to provide complementary information for structural identification using the following conditions: mass range, *m*/*z* 100–1500; nebulizer gas (gas 1) of 55 psi; heater gas (gas 2) of 55 psi; curtain gas of 35; ion spray voltage of 7 eV; turbo spray temperature (TEM) of 550 °C; declustering potential (DP) of 60 V for MS; declustering potential (DP) of 100 V for MS/MS; collision energy (CE) of 10 for MS; collision energy (CE) of 35 and collision energy spread (CES) of 15 for MS/MS; nitrogen was kept as the nebulizer and auxiliary gas. The data were acquired and analyzed using PeakView™ software, and the accurate mass and composition for the precursor and fragment ions were analyzed. Chromatographic peaks in the samples were identified by accurate mass information and retention times of targeted reference substances. Peak areas values were obtained using the XIC Manager in PeakView™ software. Extracted ion chromatograms (XICs) were automatically generated for each targeted analyte and compared against a user-defined threshold.

### 3.3. Hemostatic Assays

#### 3.3.1. Sample Preparation and Animals

SPF Wistar rats of both sexes weighing 220–250 g were purchased from the experimental animal center of Nanjing University of Chinese Medicine (Nanjing, China). All animal experiments were approved by the Animal Ethics Committee of Nanjing University of Chinese Medicine, and were carried out according to the Guidelines for the Care and Use of Laboratory Animals. Rats were housed in a conventional animal facility with free access to standard laboratory chow and tap water and were allowed to acclimatize for at least one week before experiment. Rats fasted overnight with free access to water before experimentation. The FPC-N samples were ground into fine powders and were uniformly dispersed in the 0.5% sodium carboxymethyl cellulose (CMC-Na) for animal tests.

#### 3.3.2. Experimental Procedure

The animals were divided randomly into nine groups with eight animals in each group. The negative control group was given blank solvent and the positive control group was treated with Yunnan Baiyao (YNBY, 300 mg/kg). The test groups 3–9 were treated with FPC, FPC-4, FPC-8, FPC-10, FPC-12, FPC-15, and FPC-20 (300 mg/kg) respectively through oral gavage once a day. After the fifth administration, blood was obtained from common carotid aortas. Hemostatic tests (BT, APTT, PT, TT, FIB, ELT, and platelet aggregation) were conducted according to kit instructions and the methods described in previous literatures [20,21,22].

### 3.4. Data Analysis

#### 3.4.1. Principal Component Analysis (PCA)

As a standard data reduction technique, PCA extracts data, removes redundant information, and visualizes the main relationships that exist among a large number of variables in terms of a smaller number of potential factors without losing much information [23]. In this study, PCA was performed on the quantitative hemostatic parameters of FPC-N that were obtained from hemostatic assays using SPSS 16.0 statistics software (SPSS for Windows 16.0, SPSS Inc., Chicago, IL, USA).

#### 3.4.2. Canonical Correlation Analysis (CCA)

The basic idea of CCA is to study the correlation between two sets of variations [24]. CCA can reveal and catch the most information between them [25]. CCA was herein used for the spectrum-effect relationships between the peak areas in HPLC-MS/MS XICs and the quantitative hemostatic parameters using SPSS 16.0 statistical software (SPSS for Windows 16.0, SPSS Inc.).

### 3.5. Isolation and Confirmation of the Predicted Active Peaks

#### 3.5.1. Extraction and Isolation

FPC-12 powders (25 g) were mixed with 1 L of methanol and sonicated for 1 h at 70 °C. This step was then repeated twice for complete extraction. The total extracts were combined, filtered, and evaporated with a rotary evaporator. The concentrated solution (25 mL) was subjected to C18 CC and eluted stepwise individually with 1 L of 20%, 40%, 60%, 80%, or 100% methanol. Then the eluent was collected at 400 mL intervals and analyzed by HPLC. The eluents with the same compositions were combined according to the HPLC analysis results. The pooled fractions were tested for coagulation and platelet aggregation assays. Finally, the predicted and active peaks (eluted with 80% methanol) were further applied to CC on a Sephadex LH-20 column (1200 mm × 10 mm i.d.) with dichloromethane-methanol (1:1, *v*/*v*). To isolate each compound from the corresponding fractions, HPLC analysis was performed on a Waters e2695 Separations Module system equipped with a Photodiode Array Detector. Dubhe C18 column (250 mm × 4.6 mm, 5.0 mm), an analytical column of reverse-phase HPLC, was eluted gradiently with a mobile phase of methanol (A)/0.1% formic acid (B) (0–10 min: 48% A; 10–15 min: 48%–60% A; 15–25 min: 60% A; 25–30 min: 60%–80% A; 30–40 min: 80% A) at a flow rate of 1 mL/min while maintaining the column temperature at 30 °C.

#### 3.5.2. Structural Identification

NMR (^1^H-NMR, ^13^C-NMR, HSQC, HMBC, COSY, and DEPT) spectra for the compound were recorded using NMR spectrometer systems (Bruker Avance DRX-500, Zurich, Switzerland) with DMSO-*d*_6_ as solvents, operated at 500 MHz. The chemical shifts were reported in ppm, and coupling constants (*J*) were in Hz.

#### 3.5.3. Hemostatic Activity Verification

Hemostatic activity of the compound was verified by pro-coagulation and plasma platelet aggregation assays *in vitro*. The compound was dissolved in DMSO to prepare final concentrations of 0, 1, 10, or 100 μM for platelet aggregation assay and 0, 2, 20, or 200 μM for plasma pro-coagulation assay. Etamsylate (Etam) and haemocoagulase (Haem) were selected as positive controls, and 2% DMSO was used as the blank control (Cont). ADP, collagen, and thrombin (final concentrations: 0.05 μmol/L, 0.03 μg/mL, and 0.01 u/mL, respectively) were selected as agonists for the platelet aggregation test. All measurements were determined according to kit instructions and the methods described in previous literature [26].

#### 3.5.4. Application to the Analysis of PCC Samples

The chemical compositions of PCC extract were identified using HPLC, as described in Section 3.5.1. The PCC extract solution was prepared as described in the Pharmacopoeia of the People’s Republic of China [1]. In brief, accurately weighed powders (0.5 g) were suspended in 50 mL of methanol, and sonicated for 30 min.

## 4. Conclusions

Spectrum-effect relationship was established first by combining HPLC-MS/MS fingerprints of FPC-N with hemostatic tests to reveal the potential bioactive compounds responsible for the tested hemostatic activity. A new hemostatic compound, which was generated after the flavonoid extract of PC was heated, was discovered and named cecarbon, which may be valuable for gaining insight into the unique herb processing practice in TCM. This study provides an available reference model for tracing the active compounds of other herbs that have stronger bioactivities after processing.

## Supplementary Materials

Supplementary materials can be accessed at: http://www.mdpi.com/1420-3049/20/09/16970/s1.

## Abbreviations

*Platycladi Cacumen* (PC); *Platycladi Cacumen Carbonisatus* (PCC); base peak chromatograms (BPC); high-performance liquid chromatography-mass spectroscopy/mass spectroscopy (HPLC-MS/MS); heating products of total flavonoids in *Platycladi Cacumen* (FPC-N); nuclear magnetic resonance (NMR); mass spectroscopy (MS); Traditional Chinese Medicine (TCM); activated partial thromboplastin time (APTT); prothrombin time (PT); thrombin time (TT); fibrinogen content (FIB); rat-tail bleeding time (BT); euglobulin lysis time (ELT); the flavonoid extract from *Platycladi Cacumen* (FPC); FPC was heated for 4 min (FPC-4), 8 min (FPC-8), 10 min (FPC-10), 12 min (FPC-12), 15 min (FPC-15), or 20 min (FPC-20); principal component analysis (PCA); canonical correlation analysis (CCA);column chromatography (CC); ultraviolet spectroscopy (UV); extracted ion chromatograms (XICs); Yunnan Baiyao (YNBY); Etamsylate (Etam); Haemocoagulase (Haem); blank control (Cont).

## References

[1] Commission C.P. (2010). The Pharmacopoeia of the People’s Republic of China.

[2] Kim T.H., Li H., Wu Q., Lee H.J., Ryu J.H. (2013). A new labdane diterpenoid with anti-inflammatory activity from *Thuja orientalis*. J. Ethnopharmacol..

[3] Lu Y.H., Liu Z.Y., Wang Z.T., Wei D.Z. (2006). Quality evaluation of *Platycladus orientalis* (L.) Franco through simultaneous determination of four bioactive flavonoids by high-performance liquid chromatography. J. Pharm Biomed Anal..

[4] Pelter A., Warren R., Hameed N., Khan N.U., Ilyas M., Rahman W. (1970). Biflavonyl pigments from *Thuja orientalis* (Cupressaceae). Phytochemistry.

[5] Khabir M., Khatoon F., Ansari W.H. (1985). Phenolic constituents of *Platycladus orientalis*. Curr. Sci..

[6] Liu C., Liu J., Zhang L., Li S.F., Zhang L.N., Ding A.W., Yu B. (2014). Comparison on hemostasis of *Platycladi cacumen* before and after processing on blood heat and hemorrhage syndrome rat model. Chin. Tradit. Herb. Drugs.

[7] Wu H.E., Zhen H.H., Wei Z.Y., Chen C.L. (2009). Determination of quercetin and kaempferol in *Platycladus orientalis* carbonisatus by RP-HPLC. Lishizhen Med. Mater. Med. Res..

[8] Sun L.L., Yang S.B., Jiang B., Zhong F.X., Shi D.H. (2006). The effect of processing on *Platycladi cacumen* chemical components. Chin. Tradit. Patent Med..

[9] Figueiredo-González M., Cancho-Grande B., Boso S., Santiago J.L., Martínez M.C., Simal-Gándara J. (2013). Evolution of flavonoids in Mourton berries taken from both bunch halves. Food Chem..

[10] Li Z.F., Wang Y.W., Ouyang H., Lu Y., Qiu Y., Feng Y.L., Jiang H.L., Zhou X., Yang S.L. (2015). A novel dereplication strategy for the identification of two new trace compounds in the extract of Gastrodia elata using UHPLC/Q-TOF-MS/MS. J. Chromatogr. B.

[11] Peng Y., Zhao L., Lin D.J., Liu Y., Zhang M., Song S.J. (2015). The chemical constituents’ determination of the different processed products of Anemarrhena asphodeloides Rhizomes by high-performance liquid chromatography quadrupole time-of-flight mass spectrometry (HPLC-Q-TOF-MS/MS). Biomed. Chromatogr..

[12] Yang H., Lee D.Y., Kang K.B., Kim J.Y., Kim S.O., Yoo Y.H., Sung S.H. (2015). Identification of ginsenoside markers from dry purified extract of Panax ginseng by a dereplication approach and UPLC-QTOF/MS analysis. J. Pharm. Biomed. Anal..

[13] Lippi G., Favaloro E.J. (2013). Laboratory hemostasis: milestones in Clinical Chemistry and Laboratory Medicine. Clin. Chem. Lab. Med..

[14] Song Z.L., Hashi Y.K., Sun H.Y., Liang Y., Lan Y.X., Wang H., Chen S.Z. (2013). Simultaneous determination of 19 flavonoids in commercial trollflowers by using high-performance liquid chromatography and classification of samples by hierarchical clustering analysis. Fitoterapia.

[15] Shaw L.H., Chen W.M., Tsai T.H. (2013). Identification of Multiple Ingredients for a Traditional Chinese Medicine Preparation (Bu-yang-huan-wu-tang) by Liquid Chromatography Coupled with Tandem Mass Spectrometry. Molecules.

[16] Tutanc M., Arica V., Motor S., Basaralan F., Erden E.S., Ozturk O.H., Zararsiz I., Aydin M. (2012). Effects of erdosteine on hemostasis: An experimental study. Hum. Exp. Toxicol..

[17] Song Q.L., Wang S.S., Zhao W.J. (2012). Total steroidal alkaloids from Veratrum patulum L. Inhibit platelet aggregation, thrombi formation and decrease bleeding time in rats. J. Ethnopharmacol..

[18] Chen Y.Q., Yu H.L., Wu H., Pan Y.Z., Wang K.L., Liu L.P., Jin Y.P., Zhang C.C. (2015). Tracing novel hemostatic compounds from heating products of total flavonoids in *Flos sophorae* by spectrum-effect relationships and column chromatography. J. Sep. Sci..

[19] Kimura H., Tokuyama S., Ishihara T., Ogawa S., Yokota K. (2015). Identification of new flavonol *O*-glycosides from indigo (*Polygonum tinctorium* Lour) leaves and their inhibitory activity against 3-hydroxy-3-methylglutaryl-CoA reductase. J. Pharm. Biomed. Anal..

[20] Dejana E., Callioni A., Quintana A., Gaetano G. (1979). Bleeding time in laboratory animals. II—A comparison of different assay conditions in rats. Thromb. Res..

[21] Roshal M., Shaz B.H., Hillyer C.D., Roshal M., Abrams C.S. (2013). Laboratory Techniques in Fibrinolysis Testing. Transfusion Medicine and Hemostasis-Clinical and Laboratory Aspects.

[22] Mustard J.F., Perry D.W., Ardlie N.G., Packham M.A. (1972). Preparation of suspensions of washed platelets from humans. Br. J. Hameatol..

[23] Zheng Q.F., Zhao Y.L., Wang J.B., Liu T.T., Zhang B., Gong M., Li J.Y., Liu H.H., Han B., Zhang Y.M. (2014). Spectrum-effect relationships between UPLC fingerprints and bioactivities of crude secondary roots of *Aconitum carmichaelii Debeaux* (Fuzi) and its three processed products on mitochondrial growth coupled with canonical correlation analysis. J. Ethnopharmacol..

[24] Gumus E., Kursun O., Sertbas A., Ustek D. (2012). Application of canonical correlation analysis for identifying viral integration preferences. Bioinformatics.

[25] Liu X., Wang X.L., Wu L., Li H., Qin K.M., Cao H., Pei K., Liu T., Cai B.C. (2014). Investigation on the spectrum-effect relationships of Da-Huang-Fu-Zi-Tang in rats by UHPLC-ESI-Q-TOF-MS method. J. Ethnopharmacol..

[26] Liu L., Duan J.A., Tang Y., Guo J.M., Yang N.Y., Ma H.Y., Shi X.Q. (2012). Taoren-Honghua herb pair and its main components promoting blood circulation through influencing on hemorheology, plasma coagulation and platelet aggregation. J. Ethnopharmacol..

