# Supplementary material for: A Novel Reduplicate Strategy for Tracing Hemostatic Compounds from Heating Products of the Flavonoid Extract in Platycladi cacumen by Spectrum-Effect Relationships and Column Chromatography
Source: Molecules. 2015 Sep 17;20(9):16970–86. doi: 10.3390/molecules200916970 (PMC6332512; doi:10.3390/molecules200916970)
Supplement: Supplementary file 1 [file molecules-20-16970-s001.pdf]

Supplementary Materials

Table S1. Mark compounds contents in the flavonoid extract in Platycladi Cacumen (FPC).

Samples	Content (%, *n* = 3)			
	**Myricetrin**	**Kaempferol-3-*O*-rhamnoside**	**Quercitrin**	**Amentoflavone**
FPC	20.22	10.45	44.74	14.41

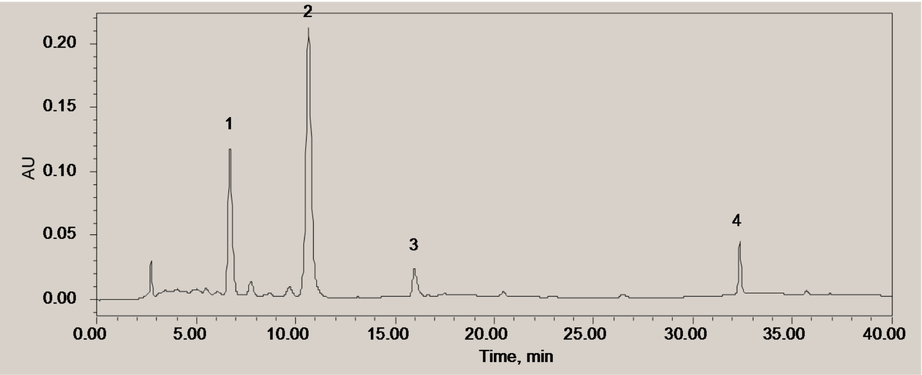

Figure S1. HPLC chromatogram of sample FPC. Four peaks were identified by comparing with the standard substance: myticetrin (1), quercetrin (2), kaempferol-3-*O*-rhamnoside (3), amentoflacone (4).

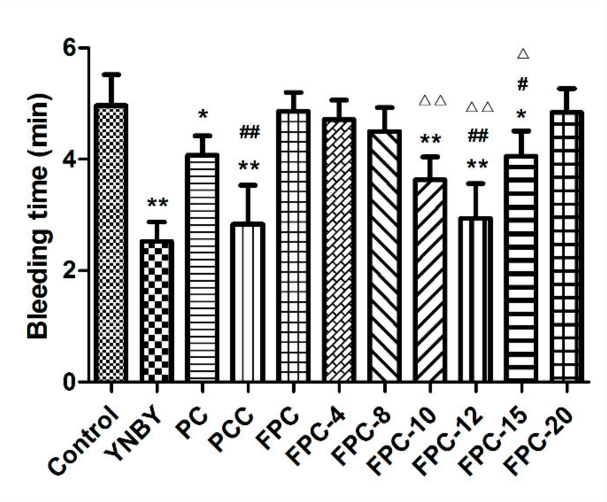

Figure S2. Effect of PC, PCC and FPC-N on haemostasis in rat was analysed by measuring the bleeding time after removal of 2 mm of tail tip. Data are represented as mean ± SD of 8 animals. The significance between control and treated groups, PC group and the other treated groups, FPC group and the other FPC-N groups, and the *p* values were calculated by one-way analysis of variance. As compared to control group, ** $p < 0.01$, * $p < 0.05$; as compared to PC group, $^{\#\#}$ $p < 0.01$, $^{\#}$ $p < 0.05$; as compared to FPC group, $^{\Delta\Delta}$ $p < 0.01$, $^{\Delta}$ $p < 0.05$.
